# High Prevalence of *Methanobrevibacter smithii* and *Methanosphaera stadtmanae* Detected in the Human Gut Using an Improved DNA Detection Protocol

**DOI:** 10.1371/journal.pone.0007063

**Published:** 2009-09-17

**Authors:** Bédis Dridi, Mireille Henry, Amel El Khéchine, Didier Raoult, Michel Drancourt

**Affiliations:** Unité de Recherche sur les Maladies Infectieuses et Tropicales Emergentes UMR CNRS 6236 IRD 3R198, IFR 48, Faculté de Médecine, Université de la Méditerranée and Pôle des Maladies Infectieuses, Assistance Publique-Hôpitaux de Marseille, Marseille, France; University of Würzburg, Germany

## Abstract

**Background:**

The low and variable prevalence of *Methanobrevibacter smithii* and *Methanosphaera stadtmanae* DNA in human stool contrasts with the paramount role of these methanogenic *Archaea* in digestion processes. We hypothesized that this contrast is a consequence of the inefficiencies of current protocols for archaeon DNA extraction. We developed a new protocol for the extraction and PCR-based detection of *M. smithii* and *M. stadtmanae* DNA in human stool.

**Methodology/Principal Findings:**

Stool specimens collected from 700 individuals were filtered, mechanically lysed twice, and incubated overnight with proteinase K prior to DNA extraction using a commercial DNA extraction kit. Total DNA was used as a template for quantitative real-time PCR targeting *M. smithii* and *M. stadtmanae* 16S rRNA and *rpo*B genes. Amplification of 16S rRNA and *rpo*B yielded positive detection of *M. smithii* in 95.7% and *M. stadtmanae* in 29.4% of specimens. Sequencing of 16S rRNA gene PCR products from 30 randomly selected specimens (15 for *M. smithii* and 15 for *M. stadtmanae*) yielded a sequence similarity of 99–100% using the reference *M. smithii* ATCC 35061 and *M. stadtmanae* DSM 3091 sequences.

**Conclusions/Significance:**

In contrast to previous reports, these data indicate a high prevalence of the methanogens *M. smithii* and *M. stadtmanae* in the human gut, with the former being an almost ubiquitous inhabitant of the intestinal microbiome.

## Introduction

*Archaea* are environmental organisms that are associated with the mucosa in mammals [1]–[6]. In humans, *Archaea* are associated with the vaginal, oral, and intestinal mucosa [1]–[6]. Because of the fastidious nature of these strict anaerobes, most current knowledge about the archaeal flora of mammals is derived from DNA-based analyses. In the human gut, methanogenic *Archaea* metabolize major fermentation products, such as alcohols, short chain organic acids, carbon dioxide (CO_2_), and hydrogen (H_2_) [7]. Until recently, the diversity of gut methanogens was thought to be limited to two species: *Methanobrevibacter smithii*, the most abundant methanogenic *Archaea* found in the human gut [1]–[6], [8]–[10], and *Methanosphaera stadtmanae*, which is seldom detected in the human gut. Both species can be detected by culture and PCR-based assays targeting the 16S rRNA and *mcr*A genes [8]. Recently, the DNA of additional methanogenic *Archaea* has been detected in human stool specimens, including DNA sequences that indicate the presence of a new order of methanogenic *Archaea* [2], [11].

The predominance of *M. smithii* compared to *M. stadtmanae* can be explained by the fact that the genome of the former is more tailored to the gut environment with regards to metabolic versatility, genomic evolution ability, and persistence [9]. In the gut, *M. smithii* converts H_2_, CO_2,_ and formate into CH_4_ using carbon as the terminal electron acceptor; this redox reaction sustains anaerobic respiration, which allows for the production of ATP [12], [13]. This archaeon can also remove fermentation end products, such as methanol and ethanol, produced by other bacteria lacking a methanogenic pathway, while *M. stadtmanae* energy metabolism is limited to using hydrogen to reduce methanol to methane and is dependent on acetate as a carbon source [8]. Methanogenesis is of paramount importance in preventing the accumulation of gases and other reaction end products [9]. Accordingly, a metagenomic analysis of the gut flora in three healthy individuals found that *M. smithii* comprised up to 11.5% of the gut microorganisms [14]. However, 16S rRNA- and *mcr*A gene-based studies detected *M. smithii* with variable prevalence in less than half of the tested individuals and no *M. stadtmanae*; such results cannot reflect the actual quantity of these two *Archaea* in the human gut because of their specific association with the gut mucosa [14]. We hypothesized that the variable prevalence of *M. smithii* DNA in human stool specimens and the failure to detect *M. stadtmanae* DNA in various studies may be due to limitations in the experimental protocols and to the relatively small samples comprising three or six individuals [14], [15]. We aimed to establish an optimized protocol for the extraction and detection of archaeal DNA by exploiting the *M. smithii* and *M. stadtmanae* genome sequences [8], [10].

## Materials and Methods

### 2.1. Source of fecal samples

A series of 700 fecal specimens were prospectively collected to investigate the prevalence of *M. smithii* and *M. stadtmanae*. Stool specimens were stored at −20°C until used. A random subset of 50 specimens was further used to compare the DNA extraction method developed herein with a reference method. Only one stool specimen was collected per person, and for each person, sex, age, and presence of an enteric pathogen in the stool specimen responsible for diarrhea were documented. This study was approved by the local Ethics Committee.

### 2.2. Fecal DNA isolation

Approximately 1 gram of specimen was suspended in 10 ml of saline buffer and filtered through a Fecal Specimen Filtration Vial (Orion Diagnostica–Fumouze-Division Diagnostics, Levallois Perret, France). Aliquot of the filtrate (250 µl) was transferred into a sterile screw-cap Eppendorf tube containing 0.3 g of acid-washed glass beads (≤106 µm, Sigma, Saint-Quentin Fallavier, France) and shaken to achieve mechanical lysis in a FastPrep BIO 101 apparatus (Qbiogene, Strasbourg, France) at level 6.5 (full speed) for 90 s. The supernatant was collected and incubated overnight at 56°C with 180 µl of T1 Buffer from the NucleoSpin® Tissue Mini Kit (Macherey Nagel, Hoerdt, France) and 25 µl of proteinase K (20 mg/ml). After a second cycle of mechanical lysis as described above, the filtrate was incubated for 15 min at 100°C. Total DNA was then extracted using the NucleoSpin® Tissue Mini Kit, according to the manufacturer's procedure. Extracted DNA was eluted with 100 µl of elution buffer and stored at −20°C until used. A mock extraction performed using 250 µl of sterile water was used as a negative control for each batch of DNA extractions.

### 2.3. Real-time quantitative PCR assays

The targeted genes, probes, primer sequences (Eurogentec, Seraing, Belgium), and PCR product sizes for the four real-time PCR assays developed in this study are summarized in Table 1. The sequences of the four probes (Applied Biosystems, Courtaboeuf, France) and four primer pairs were designed using the *M. smithii* ATCC 35061 complete genome (GenBank accession number CP000678) and the *M. stadtmanae* DSM 3091 complete genome sequence (GenBank accession number NC 007681) (both exhibiting only one copy of the 16S rRNA gene and one copy of the *rpo*B gene) via the online Primer 3 program (http://biotools.umassmed.edu/bioapps/primer3_www.cgi). The specificities of the PCR primers and probes for the *M. smithii* ATCC 35061 and *M. stadtmanae* DSM 3091 16S rRNA and *rpo*B genes were verified *in silico* using the BLAST program at NCBI (http://www.ncbi.nlm.nih.gov/BLAST). The specificity was further experimentally ensured by incorporating the DNA extracted from 43 bacterial species representative of common gut inhabitants and enteric pathogens, including *M. stadtmanae* and *M. smithii* as positive controls (Table S2), in the real-time PCR protocol reported below. The primers and Taqman probes used for total bacterial real-time qPCR were adapted from the method previously described by Palmer and collaborators [16]. Real-time PCR assays were performed with a MX3000™ system (Stratagene, Amsterdam, The Netherlands) using the QuantiTect Probe PCR Kit (Qiagen, Courtaboeuf, France) with 5 pmol of each primer, probe labeled with FAM or VIC, and 5 µl of DNA (about 2 µg of total DNA) in a final volume of 25 µl. Positive controls (DNA extracted from *M. smithii* DSM861 and *M. stadtmanae* DSM 3091 strains, German Collection of Microorganisms and Cell Cultures, Braunschweig, Germany), an extraction control, no-template controls, and a quantification scale from 10^−7^ to 10 target copies were utilized in each reaction plate. The 16S rRNA and *rpo*B gene copies were quantified in a duplex assay, and total bacteria were used in simplex real-time qPCR as a bacterial DNA extraction control. The PCR amplification program was 95°C for 15 min, followed by 45 cycles of 95°C for 30 s and 60°C for 1 min. All DNA samples were tested in duplicate. Results are expressed as the number of 16S rRNA and *rpo*B copies per gram of feces. Plasmid quantification was carried out by inserting a chimeric nucleotide fragment into the pCR II plasmid (TA cloning Kit Dual Promoter (pCRII) Invitrogen, Carlsbad, CA, USA), as previously described [17]. The chimeric fragment contained real-time PCR targets for *M. smithii* and *M. stadtmanae* 16S rRNA and *rpo*B genes, together with a universal 16S rRNA bacterial target gene adapted from the *Streptomyces sudanensis* strain SD504 sequence (GenBank accession number EF515876) as an internal control to monitor the absence of PCR inhibition.

**Table 1:** Real-time PCR primers and probes sequences for detecting *M. smithii* and *M. stadtmanae* 16S rRNA and *rpo*B genes and for detecting all bacteria (*Totbact*).

Organism	Assay	Primer/probe name and sequence (5′-3′)	Dye	Product size (bp)
*M. smithii*	16S rRNA	Smit.16S-740F, 5′-CCGGGTATCTAATCCGGTTC-3′		123
		Smit.16S-862R, 5′-CTCCCAGGGTAGAGGTGAAA-3′		
		Smit.16S FAM, 5′-CCGTCAGAATCGTTCCAGTCAG-3′	FAM (MGB)	
	*rpoB*	Ms_*rpo*BF, 5′-AAGGGATTTGCACCCAACAC-3′		70
		Ms_*rpo*BR, 5′-GACCACAGTTAGGACCCTCTGG-3′		
		Ms_*rpo*BVIC, 5′-ATTTGGTAAGATTTGTCCGAATG-3′	VIC (MGB)	
*M. stadtmanae*	16S rRNA	Stadt_16SF, 5′-AGGAGCGACAGCAGAATGAT-3′		97
		Stadt_16SR, 5′-CAGGACGCTTCACAGTACGA-3′		
		Stadt_16SFAM, 5′-TGAGAGGAGGTGCATGGCCG-3′	FAM (Taqman)	
	*rpoB*	stadt_*rpo*BF, 5′-TGCTTGGTATTTGTGCTGGA-3′		95
		stadt_*rpo*BR, 5′-TCCAAGAGCCTGTTTTGTCA-3′		
		Stadt_*rpo*BVIC, 5′-CACCAAGGAACACAATGGAGGC-3′	FAM (Taqman)	
All *bacteria*	*Totbact*	Bact 8FM, 5′-AGAGTTTGATCMTGGCTCAG-3′		327
		Bact515R, 5′-TTACCGCGGCKGCTGGCAC-3′		
		Bact338K, 5′-CCAKACTCCTACGGGAGGCAGCAG-3′	VIC (Taqman)	

### 2.4. Sequencing

To sequence the *M. smithii* and *M. stadtmanae* DNA in human feces, real time-PCR products of the 16S rRNA gene were sequenced for 30 randomly chosen specimens using the primers Smit.16S-740F, Smit.16S-862R, Stadt_16SF and Stadt_16SR (Table 1). PCR products were purified and sequenced using the BigDye Terminator 1.1 Cycle Sequencing kit and the 3130 genetic analyzer (Applied Biosystems). Negative controls were employed for each assay. Sequences were analyzed using the Seqscape program (Applied Biosystems), and similarity values were determined using the online BLAST program at NCBI.

### 2.5. Protocol sensitivity

To monitor the sensitivity of our extraction protocol, three stool specimens presenting low loads of *M. smithii* 16S rRNA and *rpo*B genes supplemented with 100 µl of *M. smithii* DSM 861 suspension were used. For all specimens in both the 16S rRNA and *rpo*B assays, the sensitivity was calculated by subtracting the gene copy number present before addition of *M. smithii* to that obtained after and then dividing by the gene copy number obtained for 100 µl of a pure suspension of *M. smithii*. The protocol sensitivity presented is the average of the three percentages obtained.

### 2.6. Comparison with the reference DNA extraction protocol

Total DNA was isolated from 50 stool specimens using the extraction protocol described above in parallel with the QIAamp Stool DNA mini kit protocol (Qiagen), as previously reported [14]. The latter kit was used as a gold standard, as a literature review indicated that this kit was used in almost all recent studies (2005–2008) dealing with DNA extraction from animal and human stools. We then compared the numbers of *M. smithii* 16S rRNA and *rpo*B gene copies per gram of stool detected by each DNA extraction protocol. Numerical data were analyzed using EPIINFO version 3.4.1 software (Centers for Disease Control and Prevention, Atlanta, GA). *P* values were used to assess statistical significance when comparing the two methods and were calculated using the non-parametric Kruskal-Wallis test for two groups. A *P* value<0.05 was considered to be significant.

## Results

### 3.1. Stool specimens

Stool specimens were collected from 700 persons [408 males (aged 1 day to 95 years) and 292 females (aged 1 month to 98 years)] (Table S1). The stool specimens of 643 of these patients had been submitted for the identification of enteric pathogens; the remaining 57 stool specimens had been submitted for the determination of *Staphylococcus aureus* and *Salmonella* spp. intestinal carriage in healthy adults without diarrhea and no history of enteric disease in the past six months. No enteric pathogens were identified in the healthy adults, whereas enteric pathogens were cultured from 4 of the remaining 643 patients, including *Salmonella enterica* in 2 patients, *Staphylococcus aureus* in 1 child, and *Shigella sonnei* in 1 patient (Table S1).

### 3.2. Real-time PCR

*In-silico* analysis using the online BLAST program at NCBI indicated that the *M. smithii* and *M. stadtmanae* real-time PCR systems were specific for the two species. These data were experimentally confirmed by the fact that the systems did not detect the DNA extracted from 43 other enteric organisms in the presence of positive controls, with the exception of the homologous methanogenic species. Negative results were obtained for all negative controls in the assay. Our protocol demonstrated an extraction efficiency of 89.82% for the 16S rRNA assay and 94.04% for the *rpo*B assay. In the present study, 670/700 stool specimens were positive for the *M. smithii* 16S rRNA or *rpo*B genes (95.7%), including 133 specimens that were positive for the 16S rRNA gene only. The 16S rRNA and the *rpo*B gene copy numbers varied from 10.9−1.45×10^11^ and from 2.22−2.40×10^10^ copies per gram of stool, respectively (Table 2). The internal positive control was detected in 29/29 (100%) stool specimens that remained negative for *M. smithii* DNA.

**Table 2:** Mean, standard deviation, median, and maximal and minimal values of the copy numbers of *M. smithii* and *M. stadtmanae*16S rRNA and *rpo*B genes per gram of stool in the positive specimens for each assay.

	*M. smithii*		*M. stadtmanae*	
	16S rRNA assay	*rpo*B assay	16S rRNA assay	*rpo*B assay
**Minimum**	1,09E+01	2,22E+00	1,20E+01	3,79E+01
**Maximum**	1,45E+11	2,40E+10	1,45E+10	2,74E+09
**Target copy number Mean**	2,33E+09	6,46E+08	1,80E+08	6,56E+07
**Standard deviation**	1,26E+10	2,70E+09	1,25E+09	3,40E+08
**Median**	4,20E+04	1,72E+04	9,30E+03	2,66E+04

Among these 700 stool specimens, 206 (29.4%) were positive for *M. stadtmanae* DNA, including 71 that were positive for the 16S rRNA gene only. In all cases, *M. stadtmana*e DNA was detected in association with *M. smithii* DNA (Table S1). The 16S rRNA and the *rpo*B gene copy numbers varied from 12−1.45×10^10^ and from 37.9−2.74×10^9^ copies per gram of stool, respectively (Table 2). The internal control was detected in 44/44 (100%) randomly selected stool specimens that lacked *M. stadtmanae* DNA PCR product.

No statistical correlation was observed between *M. smithii*/*M. stadtmanae* detection or load and the presence of an enteric pathogen. No statistical difference was detected in *M. smithii*/*M. stadtmanae* detection and load between the 57 healthy individuals and the individuals who submitted stool samples for pathogen identification.

### 3.3. Sequencing

All negative controls lacked PCR products. Direct sequencing of the 30 16S rRNA gene PCR products yielded a BLAST similarity of 99% with the reference *M. smithii* ATCC 35061 16S rRNA gene sequence (GenBank accession NC_009515) and a BLAST similarity of 100% with the reference *M. stadtmanae* DSM 3091 16S rRNA gene sequence (GenBank accession CP000102). Interestingly, *M. smithii* DNA was detected in 2 specimens collected from children aged 1 and 2 years, with estimated numbers of 10 and 778 *M. smithii* per gram of stool, respectively. A sequence similarity of 99% compared to the *M. smithii* reference strain was obtained for both children.

### 3.4. Comparison of DNA extraction protocols

DNA extracted using the QIAamp Stool DNA mini kit was positive for *M. smithii* 16S rRNA and *rpo*B genes in 44/50 (90%) and 33/50 (66%) specimens, respectively, by quantitative real-time PCR. DNA extracted using the protocol described herein was positive for *M. smithii* 16S rRNA and *rpo*B genes in 50/50 (100%) and 49/50 (98%) specimens, respectively. Per 1 g of stool, the extraction protocol developed herein yielded 100- to 1,000-fold more gene copies than the reference extraction protocol (*P*≤10^−5^) (Table 3).

**Table 3:** Copy numbers of *M. smithii* 16S rRNA and *rpo*B genes per gram of stool obtained using the protocol reported herein *versus* those obtained via the reference Qiagen protocol for *M. smithii* DNA extraction from human stool (1).

	present extraction protocol					Qiagen extraction protocol					*P* value^(1)^
Assay	Mean	Standard Deviation	Median	Maximum	Minimum	Mean	Standard Deviation	Median	Maximum	Minimum	
16S rRNA	4.11e+09	1.23e+10	7.54e+05	7.43e+10	100	3.02e+07	1.25e+08	1.13e+03	7.20e+08	0	≤0.00001
*rpo*B	1.48e+09	3.80e+09	2.94e+06	1.65e+10	0	1.01e+07	4.11e+07	2.20e+03	2.27e+08	0	≤0.00001

*P* values were determined using the non-parametric Kruskal-Wallis test for two groups.

## Discussion

Based on a metagenomic analysis of stool samples collected from three healthy adults, *M. smithii* DNA was estimated to comprise up to 11.5% of the total human gut microbiome [14]. Such a high inoculum is in agreement with the paramount role of this archaeon in the detoxification of digestion by-products [9], but contrasts with the relatively low (<50%) rate of detection observed in studies based on either isolation or PCR-based detection of specific DNA sequences [5], [14], [16].

Here, we employed an optimized protocol for the extraction and specific PCR-based detection of *M. smithii* and *M. stadtmanae* DNA in stool samples and demonstrated that *M. smithii* DNA can be detected in the stool of almost all individuals (95.5%) and *M. stadtmanae* in 29.4% of individuals. The fact that negative results were obtained for the negative controls and the fact that all experiments were conducted under strict rules to prevent laboratory contamination, together with the recovery of original sequences, indicate that the positive detection of *M. smithii* and *M. stadtmanae* DNA achieved in this study was not due to specimen contamination. Moreover, the specificity of the real-time PCR systems developed herein was ensured by *in-silico* analysis and experimental testing using DNA extracted from common human gut inhabitants and pathogens. A high, 99–100% sequence similarity of real-time PCR products with that of reference sequences finally ensured the specificity of the real-time PCR systems herein reported. Likewise, detection of the internal positive control in all specimens negative for *M. smithii* or *M. stadtmanae* eliminated the possibility of PCR inhibition. Therefore, the data likely accurately estimate the prevalence of both *M. smithii* and *M. stadtmanae* in the population studied. Comparison of the DNA extraction protocol developed herein with the protocol previously used for metagenomic analysis of the human gut [14], [16] revealed a two to three log difference in the quantity of archaeon DNA, with greater detection achieved with the new protocol. This difference in performance was likely due to an optimized combination of previously reported lysis methods [14], [15], [18], [19]. The fact that members of the family *Methanobacteriales*, such as *M. smithii* and *M*. *stadtmanae*, have a proteinase K-resistant cell wall [20], suggests that double mechanical cell lyses using glass beads was decisive in the efficiency of DNA extraction. This fact was further illustrated by the detection of low DNA loads (Tables 2 and 3), whereas the sensitivity was measured to be ≥10^7^ cells per gram of feces in a previous study [11]. Such a huge difference in the sensitivity of *M. smithii* DNA detection may have influenced previously published data on the prevalence of *M. smithii* in stool. This protocol was also shown to be effective for DNA extraction from *Mycobacterium tuberculosis* present in stool [21].

In most previous studies, the universal 16S rRNA gene was used as a suitable target for the molecular detection of archaeal DNA present in the human and animal gut [14], [16], [18], [22]. The *mcrA* gene, which encodes the methyl-coenzyme M reductase in *M. smithii*, has also been used [15]. However, *M. stadtmanae mcr*A gene-derived primers failed to detect *M. stadtmanae* in a total of 207 individuals [11]. We therefore used the *rpo*B gene, which encodes the β subunit of RNA polymerase and is one of the core genes shared by *Bacteria* and *Archaea* [23], [24]. The *rpo*B gene has been previously used to infer phylogenetic relationships between *Archaea* and was recently demonstrated to correlate with DNA:DNA hybridization data, as well as the average nucleotide identity among prokaryotes [23], [25], [26]. In the present study, the specificity of *M. smithii* and *M. stadtmanae* detection was confirmed by evaluating two independent, universal molecular targets (16S rRNA and *rpo*B genes) using specific and highly sensitive quantitative real-time PCR. The latter gene exhibits a 12% sequence divergence between *M. smithii*, conferring a higher specificity than the 16S rRNA gene system. Moreover, sequencing of the PCR products obtained for *M. smithii* confirmed the detection specificity of the real-time PCR assays, as sequence similarity values were 99% compared to the reference *M. smithii* sequence. Likewise, sequence similarity values were 100% for *M. stadtmanae*.

Data herein presented shed new light on the biology of methanogenesis in the human gut. While breath methane measurement has a high positive predictive value, its sensitivity was lower than that of real-time molecular detection of methanogens in healthy individuals, the breath methane test being positive only for *M. smthii* inoculum >10^7^ organisms per gram of stool [27]. Indeed, breath methane measurement typically yields a prevalence of 40% [27], [28], in contrast to the 95.7% of methanogen DNA in our study. Despite the fact that molecular test, contrary to the breath methane test, detects both alive and dead methanogens, data herein reported indicate that gut methanogens are detectable in almost all individuals, regardless of them being healthy individuals or suffering from intestinal disease. Morevover, the breath methane test cannot determine the precise microbial source of methane in the gut; the fact that we detected *M. stadtmanae* in addition to *M. smithii* in about one-third of individuals, and that other methanogen DNAs have been detected in some individuals [15], [27], suggests that molecular determination of the gut methanogen flora is essential for any study dealing with the biology and the dynamics of methanogenesis in both healthy individuals and in patients presenting with intestinal disease under widespread antibiotic and dietary changes. We did not detect *M. smithii* and *M. stadtmanae* in less than 5% of individuals; whether this lack of detection reflects the limit of molecular detection or true absence of both methanogens, remains to be determined. Performing a breath methane test may help resolve this issue and give a basis to continue molecular detection of methanogens in such individuals. Our study indicates for the first time that methane production could be due to at least two archaeal species in the same individual but the *M. smithii* and *M. stadtmanae* DNA loads were highly variable among specimens. We did not collect several specimens per individual in a time-dependent manner, and therefore we cannot ascertain whether the *M. smithii* and *M. stadtmanae* DNA loads remain constant over time for an individual or vary under physiological and pathological circumstances. Interestingly, *M. smithii* DNA was detected in all stool specimens collected from 16 children younger than 2 years of age, including one specimen collected from a 2-week-old infant that was negative using the reference extraction protocol. Therefore, our results complete previous data demonstrating the transient detection of *M. smithii* DNA during the first 5–120 days of life in stool samples obtained from 7/14 children younger than one year of age [16]. These data indicate that the acquisition of *M. smithii* is an early event in newborns, resulting from maternal or environmental exposure. *M. smithii* has been detected in the vaginal flora, including that of 3/13 pregnant women [2], [16].

The DNA extraction protocol and highly specific and sensitive method of detection and quantification developed in the present study offer new tools on which to base the optimized, broad-spectrum molecular detection of *Archaea* embedded in the complex microbiomes associated with the mammalian mucosa. This protocol may aid in identifying additional species of *Archaea* and in monitoring modifications in the archaeal flora under various physiological, pathological, and therapeutic circumstances.

## Supporting Information

Table S1 The Ct values for the real-time PCR detection of M. smithii and M.stadtmanae in human stools.
(0.10 MB XLS)

Table S2 List of bacteria tested for the specificity of real-time PCR.
(0.05 MB XLS)

## References

[1] Belay N, Johnson R, Rajagopal BS, Conway de Macario E, Daniels L (1988). Methanogenic *bacteria* from human dental plaque.. Appl Environ Microbiol.

[2] Belay N, Mukhopadhyay B, Conway de Macario E, Galask R, Daniels L (1990). Methanogenic *bacteria* in human vaginal samples.. J Clin Microbiol.

[3] Miller TL, Weaver GA, Wolin MJ (1984). Methanogens and anaerobes in a colon segment isolated from the normal fecal stream.. Appl Environ Microbiol.

[4] Miller TL, Wolin MJ (1982). Enumeration of *Methanobrevibacter smithii* in human feces.. Arch Microbiol.

[5] Miller TL, Wolin MJ, de Macario EC, Macario AJ (1982). Isolation of *Methanobrevibacter smithii* from human feces.. Appl Environ Microbiol.

[6] Misawa H, Hoshi T, Kitame F, Homma M, Nakamura K (1986). Isolation of an antigenically unique methanogen from human feces.. Appl Environ Microbiol.

[7] McNeil NI (1984). The contribution of the large intestine to energy supplies in man.. Am J Clin Nutr.

[8] Fricke WF, Seedorf H, Henne A, Kruer M, Liesegang H (2006). The genome sequence of *Methanosphaera stadtmanae* reveals why this human intestinal archaeon is restricted to methanol and H2 for methane formation and ATP synthesis.. J Bacteriol.

[9] Samuel BS, Gordon JI (2006). A humanized gnotobiotic mouse model of host-archaeal-bacterial mutualism.. Proc Natl Acad Sci U S A.

[10] Samuel BS, Hansen EE, Manchester JK, Coutinho PM, Henrissat B (2007). Genomic and metabolic adaptations of *Methanobrevibacter smithii* to the human gut.. Proc Natl Acad Sci U S A.

[11] Scanlan PD, Shanahan F, Marchesi JR (2008). Human methanogen diversity and incidence in healthy and diseased colonic groups using *mcr*A gene analysis.. BMC Microbiol.

[12] Deppenmeier U (2002). The unique biochemistry of methanogenesis.. Prog Nucleic Acid Res Mol Biol.

[13] Macy JM, Snellen JE, Hungate RE (1972). Use of syringe methods for anaerobiosis.. Am J Clin Nutr.

[14] Eckburg PB, Bik EM, Bernstein CN, Purdom E, Dethlefsen L (2005). Diversity of the human intestinal microbial flora.. Science.

[15] Mihajlovski A, Alric M, Brugere JF (2008). A putative new order of methanogenic *Archaea* inhabiting the human gut, as revealed by molecular analyses of the *mcr*A gene.. Res Microbiol.

[16] Palmer C, Bik EM, Digiulio DB, Relman DA, Brown PO (2007). Development of the Human Infant Intestinal Microbiota.. PLoS Biol.

[17] Carcopino X, Henry M, Benmoura D, Fallabregues AS, Richet H (2006). Determination of HPV type 16 and 18 viral load in cervical smears of women referred to colposcopy.. J Med Virol.

[18] Nicholson MJ, Evans PN, Joblin KN (2007). Analysis of methanogen diversity in the rumen using temporal temperature gradient gel electrophoresis: identification of uncultured methanogens.. Microb Ecol.

[19] Yu Z, Morrison M (2004). Improved extraction of PCR-quality community DNA from digesta and fecal samples.. Biotechniques.

[20] Kubota K, Imachi H, Kawaskami S, Nakamura K, Harada H (2008). Evaluation of enzymatic cell treatments for application of CARD-FISH to methanogens.. J Microbiol Meth.

[21] El Khéchine A, Henry M, Raoult D, Drancourt M (2009). Detection of *Mycobacterium tuberculosis* complex organisms in the stools of patients with pulmonary tuberculosis.. Microbiology.

[22] Saengkerdsub S, Herrera P, Woodward CL, Anderson RC, Nisbet DJ (2007). Detection of methane and quantification of methanogenic *Archaea* in faeces from young broiler chickens using real-time PCR.. Lett Appl Microbiol.

[23] Klenk HP, Zillig W (1994). DNA-dependent RNA polymerase subunit B as a tool for phylogenetic reconstructions: branching topology of the archaeal domain.. J Mol Evol.

[24] Ovchinnikov Y, Monastyrskaya GS, Gubanov VV, Guryev SO, Salomatina (1982). The primary structure of *E. coli* RNA polymerase, Nucleotide sequence of the *rpo*C gene and amino acid sequence of the beta'-subunit.. Nucleic Acids Res.

[25] Adekambi T, Shinnick TM, Raoult D, Drancourt M (2008). Complete *rpo*B gene sequencing as a suitable supplement to DNA-DNA hybridization for bacterial species and genus delineation.. Int J Syst Evol Microbiol.

[26] Puhler G, Lottspeich F, Zillig W (1989). Organization and nucleotide sequence of the genes encoding the large subunits A, B and C of the DNA-dependent RNA polymerase of the archaebacterium *Sulfolobus acidocaldarius*.. Nucleic Acids Res.

[27] Stewart JA, Chadwick VS, Murray A (2006). Carriage, quantification, and predominance of methanogens and sulfate-reducing bacteria in faecal samples.. Lett Appl Microbiol.

[28] Levitt MD, Furne JK, Kuskowski M, Ruddy J (2006). Stability of human methanogenic flora over 35 years and a review of insights obtained from breath methane measurements.. Clin Gastroenterol Hepatol.

